# Editorial

**Published:** 1839-07-27

**Authors:** 


### THE MEDICAL EXAMINER.

PHILADELPHIA, JULY 27, 1839.

We publish in this number an interesting case of cancerous disease, illustrated by a coloured lithograph, for which our readers are indebted to the liberality of Dr. Pennock. Details of cases which terminate fatally are regarded by some physicians as matters of little interest, and as useless, at least, to the great majority of practitioners. This is taking a narrow view of the subject: no description of pathological appearances is wanting in interest if it be connected with the symptoms observed during life, or with the pathological relations existing between the appearances in question and other cases of disease. Even in cancerous diseases, which are confessedly obscure, and deprived of evident symptoms, it is interesting and useful to trace the mode of development of the morbid tissue, and its connection with a general disorder of the economy. A reflecting mind is never at loss in turning such facts to a useful account, not merely as illustrating questions of abstract scientific interest, but in one way or another conducing to the improvement of the therapeutic art.

We shall continue, therefore, to lay quite as much stress on cases which terminate fatally, as on those which end by the recovery of the patient, persuaded that at least as much will be gained by the study of the former as of the latter class of observations.

